# Obesity correlated to a higher risk of acquiring periodontitis: a cross-sectional study

**DOI:** 10.12688/f1000research.53823.2

**Published:** 2022-01-25

**Authors:** Chaerita Maulani, Elza Ibrahim Auerkari, Sri Lelyati C. Masulili, Lindawati S. Kusdhany, Chiquita Prahasanti, Nurtami Soedarsono

**Affiliations:** 1 Doctoral Program, Faculty of Dentistry, Universitas Indonesia, Jakarta, 10430, Indonesia; 2 Department of Oral Biology, Faculty of Dentistry, Universitas Indonesia, Jakarta, 10430, Indonesia; 3 Department of Periodontology, Faculty of Dentistry, Universitas Indonesia, Jakarta, 10430, Indonesia; 4 Departement of Prosthodontics, Faculty of Dentistry, Universitas Indonesia, Jakarta, 10430, Indonesia; 5 Departement of Periodontology, Faculty of Dentistry, Universitas Airlangga, Surabaya, 60132, Indonesia

**Keywords:** Body mass index, obesity, periodontitis

## Abstract

Background: The present study aimed to investigate the correlation between obesity and periodontitis, among other risk factors for periodontitis.

Methods:  In total, 262 Indonesian male and female subjects were analysed for body mass index (BMI), oral hygiene, plaque index, and clinically evaluated periodontitis. Statistical analysis was performed using Spearman tests and Pearson chi-square tests to estimate the correlation between BMI and periodontitis. Multivariate binary logistic analysis was conducted between covariate and periodontitis. P<0.05 was considered as statistically significant.

Results: The prevalence of obesity was 48.47%. There were positive correlations between BMI and periodontal status for healthy-mild periodontitis, moderate, and severe periodontitis respectively. BMI and periodontitis crude odds ratio (OR) = 2.31 (95% CI 1.41-3.78); p < 0.05, adjusted OR of BMI among other variables, was 1.88 (95%CI 1.05-3.37); p < 0.05. Exploration of the ROC curve found a BMI cut off point of 24.785 kg/m2.

Conclusion: Obesity by BMI measurement of ≥ 25kg/m2 correlated to a higher risk of acquiring periodontitis compared to normal-weight individuals.

## Abbreviations

AUC: Area under curve

BMI: Body mass index

BOP: Bleeding on probing

CAL: Clinical attachment loss

CI: Confidence of interval

COPD: Chronic obstructive pulmonary disease

IL: Interleukin

IQR: Interquartile range

OHI: Oral hygiene index

OR: Odds ratio

PBI: Papilla bleeding index

PD: Pocket Depth

PI: Plaque index

ROC: Receiver operating characteristic

TNF-α: Tumor necrosis factor alpha

UNC-15: University of North Carolina-15

## Introduction

Periodontitis is a chronic inflammatory disease which affected periodontal apparatus. Periodontitis shares many common risk factors with other chronic diseases such as hypertension, cardiovascular disease, and type 2 diabetes mellitus. Common risk factors include smoking, alcohol consumption, and obesity. The underlying mechanisms related to obesity are thought to contribute to inflammation and thereby to chronic disease. ^1^ The evidence showed a potential bidirectional relationship between obesity and periodontitis. Obesity act as systemic factor affecting periodontal health by worsening periodontal inflammation and increasing the risk of periodontitis progression. ^2^

The etiology of periodontitis derives from the series of complex interactions between pathologic microorganisms in the bacterial plaque and the host and modifications by systemic and local factors. ^3^ Susceptibility to periodontitis also affected by genetic factors which modulate individual responses to the environment and variations of the immune response. ^4^ There are many proinflammatory cytokines involved in periodontitis subjects, such as Interleukin-1 (IL-1), Interleukin-6 (IL-6), Tumor Necrosis Factor-α (TNF-α) which also correlates with proinflammatory cytokines in obese patients. ^5^^,^^6^ Adipose tissue is triglycerides storage during energy consumption and fatty acid release during starvation. Obesity modulates the host immune response by secreting several proinflammatory factors deriving from adipose tissue (adipocytes) which results in increased bone loss. ^7^ Adipose cells secrete bioactive molecules known as adipokines. Adipokines released in response to fat mass expansion induce low-grade chronic inflammation. Adiponectin secrete by adipocytes and functions as suppressing inflammation and promoting bone regeneration. Adiponectin decrease with obesity while the level of adiponectin also has inverse association with periodontitis. ^8^

Obesity has become a worldwide concern because it’s incidence has nearly tripled since 1975. In 2016 more than 1.9 billion adults 18 years and older were overweight, with 650 millions of those being obese. ^9^ The prevalence of obesity among adults over 18 years old in the U.S. was 39.8% according to a National Health and Nutrition Examination Survey conducted in 2015-2016. Asian non-Hispanic adults had the lowest prevalence (12.7%) compare with all other races and Hispanic-origin groups. ^10^

In Indonesia according to Indonesian basic health research 2018 (Riskesdas, 2018) the prevalence of obesity in Indonesian adults over 18 years old was 21.8% and the prevalence of periodontitis was 74.1%. Jakarta, the Indonesian capital city, has the second highest obesity prevalence among 34 other provinces at 29,8% whilst the prevalence of periodontitis in urban areas was 71.5%. ^11^

Obesity is usually defined by body mass index (BMI) measurement. According to the WHO, ‘overweight’ classification is set at the value of 25.0-29.9 kg/m ^2^ and ‘obesity’ defined as a BMI ≥ 30.0 kg/m ^2^. However in Asian regions BMIs can be set lower than the existing WHO cut-off point, with the overweight BMI at 22-25 kg/m ^2^ and the obesity BMI at 26-31 kg/m ^2^. ^12^ The WHO proposed classification of a weight by BMI in adult Asians for which obesity 1 is defined at 25-29.9 kg/m ^2^ and obesity 2 at ≥ 30 kg/m ^2^. ^13^

Increased BMI may be a potential risk factor for developing periodontitis. ^14^^–^^16^ On the other hand, other studies have shown obesity to be not related to the severity of periodontal disease, ^17^ but associated with tooth loss, oral hygiene and education level. ^18^ Obesity is related to adipokines that are secreted from adipose tissue which has an important role in regulating metabolic and vascular biology. ^19^ Meanwhile, as a result of chronic inflammatory state insulin resistance develops as well as oxidative stress. These factors could be implicated in the possible obesity and periodontitis association. ^20^ BMI and obesity in non-surgical periodontal therapy also appear to be independent predictors of poor response to the treatment. ^21^

The aim of this study was to ascertain the odds of having periodontitis in obese individuals among other periodontitis risk factors. We hypothesized that obesity correlated with having higher risk of periodontitis compared to normal-weight individuals.

## Methods

### Study design and participants

This cross-sectional study was conducted three subdistrict populations in Central Jakarta from July 2018 to March 2019. The subjects were recruited by consecutive sampling. The inclusion criteria were: healthy male and female participants aged 18-66 years old, having at least 14 teeth, who were willing to participate in the study and sign an informed consent. Exclusion criteria were any disease that could affect the general and oral health of the subject. We used STROBE checklist for this study.

### Ethical approval

Official permission was obtained from the corresponding authorities in DKI Jakarta which give legal permission to do the study in Central Jakarta subdistrict and ethical approval was obtained from the Dental Research Ethics Committee of the Faculty of Dentistry, Universitas Indonesia with the protocol number 070390418, and ethical approval number 38/Ethical Approval/FKGUI/V/2018.

### Data collection

#### Diagnostic criteria for obesity

Subject height was measured in centimetres and weight was assessed by a calibrated mechanical scale in kilograms. BMI was calculated by ratio weight and height squared (BMI calculated as kg/m ^2^ during data processing). Four categories of BMI defined by the WHO in accordance with the Asia-Pacific perspective were BMI <18.5 kg/m ^2^ as underweight, BMI 18-5-22.9 as normal weight, BMI 23.0-24.9 as overweight, BMI 25.0-29.9 as obesity 1, and ≥30 kg/m ^2^ as obesity 2. ^13^

#### Diagnostic criteria for periodontitis

Periodontal examination was performed using periodontal probe UNC-15. Clinical parameters of oral hygiene were measured by the simplified oral hygiene index (OHI) ^22^ and plaque index (PI). ^23^ Periodontal status was recorded by pocket depth (PD), recession, and clinical attachment loss (CAL) which measured six sites per tooth except for third molars. Measurements were made in millimetres and were rounded to the nearest whole millimetre. Bleeding on probing was recorded with papilla bleeding index (PBI) by Saxer and Muhlemann ^24^ and the number of teeth also recorded.

Patients were also categorized according to periodontal condition where CAL 5 mm and PD 6 mm were cut off measurements between mild and severe periodontitis. ^25^ Severe periodontitis was determined as CAL ≥ 5 mm at more than 18 sites and PD ≥ 6 mm in at least one site. Moderate periodontitis determined as CAL ≥ 5 mm at 9-18 sites and PD ≥ 6 mm at not more than one site. Mild periodontal status (healthy gingiva, gingivitis and mild periodontitis) was determined by CAL ≥ 5 mm at not more than eight sites and no PD ≥ 6 mm. The periodontal measurement was taken by calibrated periodontists. Analysis of inter-examiner reliability for periodontal status and plaque index was performed, and demonstrated good agreement.

The sample size was calculated for α as type I error rate (two-tailed) 0.05, type II error rate 0.20 and expected correlation coefficient 0.2, standard normal deviate for α = Zα 1.96 and standard normal deviate for β = Zβ 0.84, minimal sample size is 194. ^26^ Adding 10% for drop out or bias sample, minimal sample is 213. Null hypothesis is no effect of obesity towards the severity of periodontitis.

### Statistical analysis

The Statistical Package for the Social Sciences (SPSS version 23.0) was used to process data. If there were missing data, the samples were excluded from the study. The normality test and descriptive statistics were calculated using Shapiro-Wilk or Kolmogorov-Smirnov tests for distribution with BMI as the dependent factor. Normally distributed data were presented as mean and standard deviation, non-normally distributed data presented as medians and interquartile range (IQR), and categorical data as percentages. We analyzed the correlation between confounding factors and periodontal status with BMI in continuous data. The confounding factors and periodontitis in dichotomy was correlated with five categorical BMI. The correlation between continuous data of clinical parameter periodontal and categorical BMI was assessed. The association between healthy and mild periodontitis, moderate periodontitis, periodontitis and BMI were calculated using Kruskal Wallis tests. Dichotomy of periodontal statuses as dependent variables determined the correlation with periodontitis risk-factors including BMI. The confounding factors were controlled by multiple logistic regression. Both significant crude odds ratio and adjusted ratio were calculated to assess influenced independent variables on periodontal status (95% CI). The effect of each independent variable was measured adjusting for all variables in the model; p < 0.05 was accepted as statistically significant. The ROC was also measured to seek the specific BMI cut-off point in periodontitis (binary) within this study. We included all the subject that match the inclusion criteria until minimal number sample were sufficient. Incomplete or missing data were addressed by not included in the final data. No sensitivity analyses included in the data analyses.

## Results

**Figure 1.**
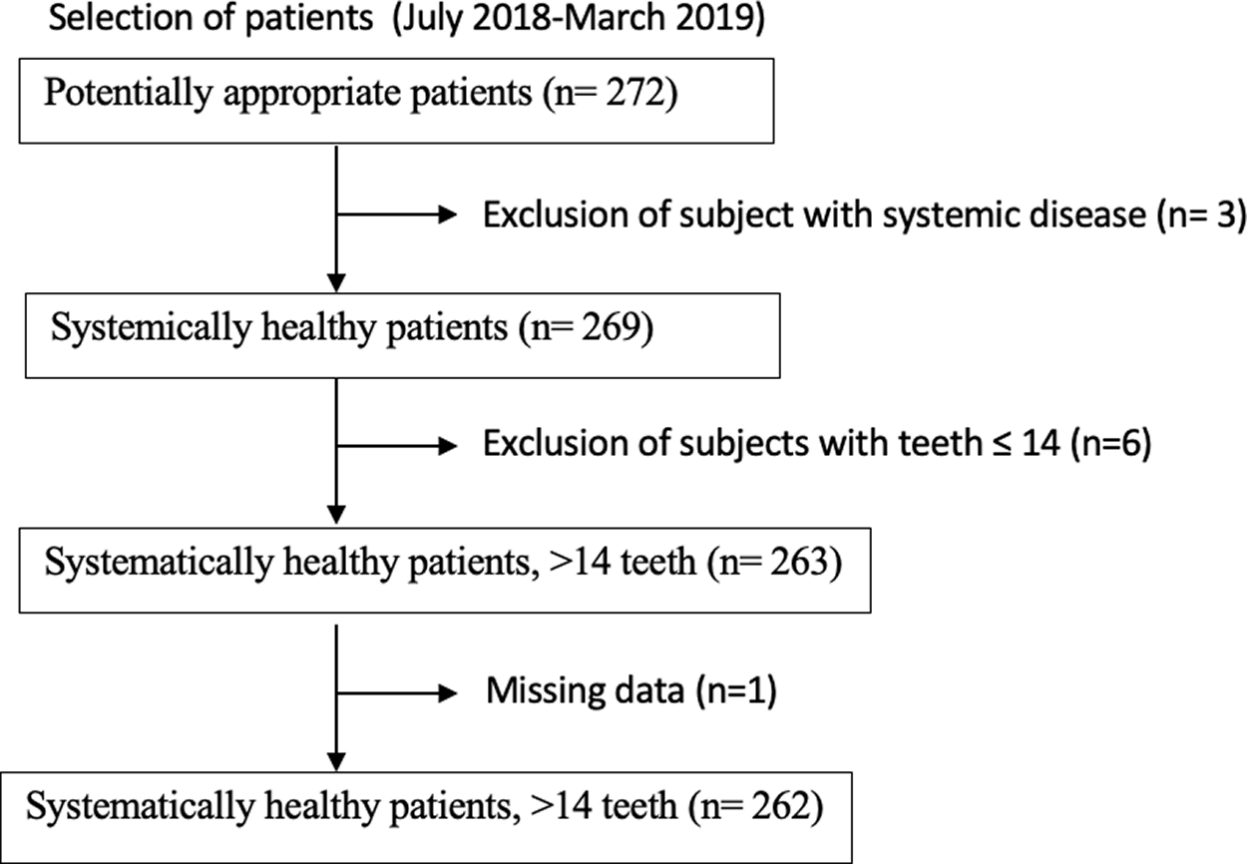
Flow diagram of the study.

A total of 272 subjects were recruited; however, some subjects were not eligible according to the inclusion criteria: such as having fewer than 14 teeth, or age. Missing data were excluded ( Figure 1). Therefore, a total of 262 subjects between the ages of 18 and 66 were included in this study. The percentage of female subjects was larger (59.9%) than male subjects (40.1%) as presented in the general profile ( Table 1). The age group of 45-54 made up two fifths of the sample size, whereas the fewest subjects were in the 25-34 years age group. Subject’s education status was mostly high school (53.4%) and the primary occupation was housewife (47.3%). The subjects were mostly non-smokers (79.4%) and non-alcohol-consumers (93.5%). 50.8% of subjects had either healthy periodontal scores or mild periodontitis, and the rest had moderate or severe periodontitis.

**Table 1.** Correlation between socio-demographic data, oral hygiene and continuous data of body mass index.

Characteristic	n	(%)	BMI (kg/m ^2^)	Correlation coefficient	P-value
Age (years), median (IQR)					0.000
18-24	49	18.7	21.83 (5.16)	0.286 ^a^ **	
25-34	28	10.7	22.61 (5.43)		
35-44	30	11.5	26.40 (7.48)		
45-54	106	40.5	25.97 (6.93)		
55-66	49	18.7	26.41 (5.71)		
Sex, mean ± SD				0.263 ^b^ **	0.000
Male	105	40.1	23.03 (6.31)		
Female	157	59.9	26.12 (6.75)		
Education, median (IQR)				0.103 ^a^	0.095
Graduate/above	38	14.5	24.08 (5.98)		
High school	140	53.4	24.23 (6.85)		
Primary/secondary/no education	84	32.1	25.45 (5.74)		
Occupation, median (IQR)				−0.270 ^a^ **	0.000
Government/private/self employed	39	14.9	25.16 (6.53)		
Housewife	124	47.3	26.41 (6.59)		
College student	19	7.3	23.84 (8.41)		
Cleaning service/security/driver/labourer	80	30.5	22.61 (5.88)		
Smoking status, median (IQR)				−0.142 ^a^ *	0.021
Non-smoker	208	79.4	25.24 (6.49)		
Smoker	54	20.6	23.01 (6.43)		
Alcohol consumption, median (IQR)				−0.017 ^a^	0.785
No alcohol consumption	245	93.5	24.77 (6.24)		
Yes	17	6.5	24.31 (5.50)		
Hypertension, mean ± SD				0.251 ^b^ **	0.000
No	188	71.8	24.54 ± 4.76		
Yes	74	28.2	27.34 ± 5.17		
Diabetes Mellitus, median (IQR)				−0.021 ^a^	0.738
No	258	98.5	24.69 (6.38)		
Yes	4	1.5	24.64 (3.98)		
Plaque Index, mean ± SD				0.082 ^b^	0.186
Slight	130	49.6	24.91 ± 5.07		
Abundance	132	50.4	25.74 ± 4.98		
Periodontal status, median (IQR)				0.208 ^a^ **	0.001
Health and Mild periodontitis	133	50.8	23.31 (6.27)		
Moderate periodontitis	54	20.6	26.17 (6.03)		
Severe periodontitis	75	28.6	26.00 (6.20)		

^a^ Spearman test.

^b^ Pearson test.

** significant < 0.01, 2-tailed.

* significant < 0.05, 2-tailed.

The socio-demographic and BMI analysis showed significant positive correlations for age, sex, hypertension, and periodontal status. Significant negative correlations were shown for occupation and smoking status (coefficient correlation −0.270, p = 0.000; coefficient correlation −0.142, p = 0.021, respectively) ( Table 1). Education, alcohol, DM, and plaque index showed no correlations with periodontal status.

The prevalence of obesity in this study was 48.47% and the highest prevalence was in BMI group 25.0-29.9 kg/m ^2^ (32.44%), then 18.5-22.9 kg/m ^2^ (29.77%). Table 2 presents the correlation between binary socio-demography data, oral hygiene, and BMI as a dependent factor. A significant positive correlation found between periodontitis and age, sex, occupation, and hypertension and while smoking showed a negative correlation. Continuous data of the clinical parameters of periodontitis and BMI is shown in Table 3. PD, CAL, and PBI were found to have significant correlations with BMI.

**Table 2.** Correlation between socio-demographic data, DM, hypertension, oral hygiene and body mass index.

Variables	Body mass index n (%)					Coefficient correlation	p-value
	<18.5	18.5-22.9	23.0-24.9	25.0-29.9	≥30.0		
	(n = 18)	(n= 78)	(n = 39)	(n = 85)	(n = 42)		
Age						0.343**	0.000
<30 years	10 (15.4)	30 (46.2)	10 (15.4)	14 (21.5)	1 (1.5)		
≥30 years	8 (4.1)	48 (24.4)	29 (14.7)	71 (36.0)	41 (20.8)		
Sex						0.267**	0.000
Male	12 (11.4)	40 (38.1)	16 (15.2)	30 (28.6)	7 (6.7)		
Female	6 (3.8)	38 (24.2)	23 (14.6)	55 (35.0)	35 (22.3)		
Education						0.053	0.397
>12 years	4 (10.3)	13 (33.3)	4 (10.3)	13 (33.3)	5 (12.8)		
≤12 years	14 (6.3)	65 (29.1)	35 (15.7)	72 (32.3)	37 (16.6)		
Occupation						0.188**	0.002
Working	10 (8.5)	44 (37.3)	18 (15.3)	33 (28.0)	13 (11.0)		
Not working	8 (5.6)	34 (23.6)	21 (14.6)	52 (36.1)	29 (20.1)		
Smoking						−0.130*	0.036
Nonuser	15 (7.2)	54 (26.0)	32 (15.4)	70 (33.7)	37 (17.8)		
User	3 (5.6)	24 (44.4)	7 (13.0)	15 (27.8)	5 (9.3)		
Alcohol						−0.131	0.618
Nonuser	16 (6.5)	73 (29.8)	37 (15.1)	79 (32.2)	40 (16.3)		
User	2 (11.8)	5 (29.4)	2 (11.8)	6 (35.3)	2 (11.8)		
Hypertension						0.196**	0.001
No	18 (9.6)	65 (31.0)	33 (15.7)	68 (32.4)	26 (12.4)		
Yes	0 (0)	13 (25.0)	6 (11.5)	17 (32.7)	16 (30.8)		
Diabetes Mellitus						0.001	0.981
No	18 (7.0)	77 (29.8)	38 (14.7)	83 (32.2)	42 (16.3)		
Yes	0 (0.0)	1 (25.5)	1 (25.0)	2 (50.0)	0 (.0)		
Periodontitis						0.219**	0.000
Mild	16 (12.0)	44 (33.1)	22 (16.5)	34 (25.6)	17 (12.8)		
Severe	2 (1.6)	34 (26.4)	17 (13.2)	51 (39.5)	25 (19.4)		
OHI						−0.027	0.664
Good (0.0-2.00)	8 (6.1)	41 (31.1)	18 (13.6)	41 (31.1)	24 (18.2)		
Poor (2.01-6.00)	10 (7.7)	37 (28.5)	21 (16.2)	44 (33.8)	18 (13.8)		
Plaque Index						0.095	0.124
Slight (0.0-1.39)	10 (7.7)	45 (34.6)	16 (12.3)	41 (31.5)	18 (13.8)		
Abundance (1.4-3.0)	8 (6.1)	33 (25.0)	23 (17.4)	44 (33.3)	24 (18.2)		

Spearman test.

**Table 3.** Correlation of clinical parameter periodontitis and BMI.

Variables, Median (IQR)	Body mass index					Correlation coefficient	p-value
	<18.5	18.5-22.9	23.0-24.9	25.0-29.9	≥30.0		
	(n = 18)	(n = 78)	(n = 39)	(n = 85)	(n = 42)		
PD ^a^ (mm)	1.49 (0.48)	1.64 (0.50)	1.67 (0.59)	1.80 (0.60)	1.74 (0.66)	0.206	0.001
CAL ^a^ (mm)	1.85 (0.65)	2.04 (1.26)	2.33 (1.24)	2.47 (1.02)	2.31 (1.00)	0.217	0.000
PBI ^a^	0.96 (0.99)	1.05 (1.04)	0.96 (1.06)	1.16 (1.04)	1.45 (1.39)	0.164	0.008
Number of teeth ^a^	25.5 (4)	26 (5)	26 (6)	25 (5)	25 (5)	−0.11	0.084
OHI ^b^ (mean ± SD)	2.11 ± 0.92	2.05 ± 0.93	2.19 ± 0.85	2.11 ± 0.88	1.96 ± 0.87	−0.023	0.715
Plaque index ^b^ (mean ± SD)	1.12 ± 0.45	1.08 ± 0.54	1.26 ± 0.53	1.17 ± 0.50	1.14 ± 0.49	0.047	0.453

^a^ Spearman tests.

^b^ Pearson tests.

**Figure 2.**
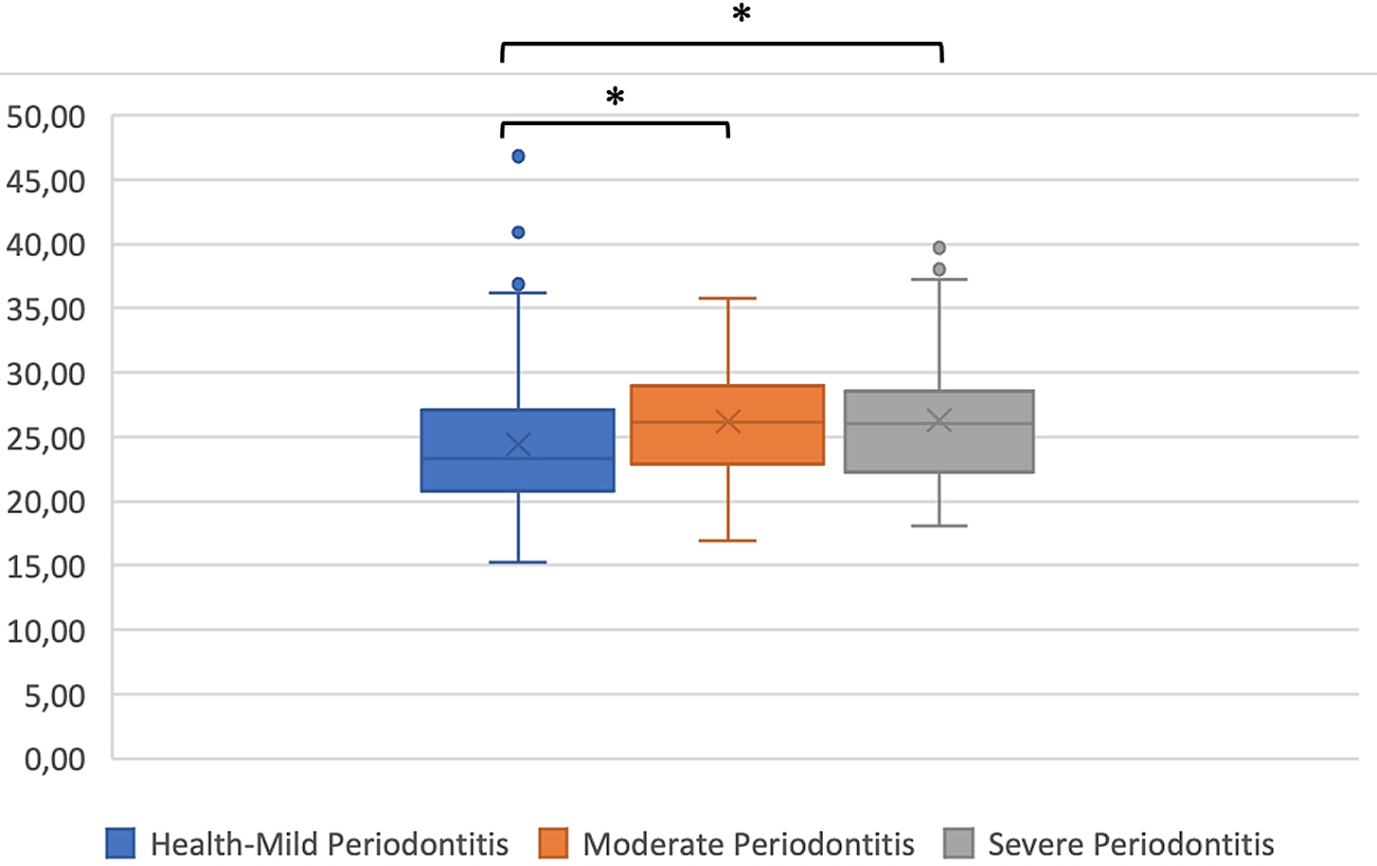
Body mass index and periodontal status; Kruskal-Wallis test *p < 0.05.

A significant positive correlation found between BMI and periodontal status. There was an association between mild periodontal status and moderate periodontitis, and between mild periodontal status and severe periodontitis were p = 0.03 and p = 0.04 respectively ( Figure 2).

Binary periodontal status categorized moderate and severe periodontitis as one group and healthy periodontal and mild periodontitis as another group. Binary periodontal status was then analyzed as a dependent factor towards the various risk factors ( Table 4). The multivariate logistic regression analysis was then performed for variables with p < 0.25 (age, sex, smoking, DM, BMI and OHIS). Diabetes mellitus status and smoking had p-values above 0.25, which were p = 0.301 and p = 0.431 respectively, but both were included in the analyses because DM and smoking status are both important risk factors in periodontal classification.

**Table 4.** Association between risk factors of periodontitis and periodontal status.

Variables	Total n (%)	Healthy periodontal & mild periodontitis n= 133	Moderate & severe periodontitis n= 129	Unadjusted		Adjusted	
				p-value	OR (95% CI)	p-value	OR (95% CI)
Age				0.000**	6.459 (3.246-12.853)	0.087	2.289 (0.888-5.900)
Young < 30 years	65 (24.8)	53 (81.5)	12 (18.5)				
Old ≥ 30 years	197 (75.2)	80 (40.6)	117 (59.4)				
Sex				0.000**	3.434 (2.037-5.789)	0.000**	6.852 (2.540-18.481)
Male	105 (40.1)	72 (68.6)	33 (31.4)				
Female	157 (59.9)	61 (38.9)	96 (61.1)				
Years of education				0.071	1.901 (0.940-3.848)	0.496	0.745 (0.320-1.737)
>12 years	39 (14.9)	25 (64.1)	14 (35.9)				
≤12 years	223 (85.1)	108 (48.4)	115 (51.6)				
Occupation				0.044	1.653 (1.012-2.701)	0.915	0.957 (0.430-2.132)
Working	118 (45)	68 (57.6)	50 (42.4)				
Not working	144 (55)	65 (45.1)	79 (54.9)				
Smoking				0.431	0.785 (0430-1.432)	0.007	3.803 (1.449-9.979)
Nonusers	208 (79.4)	103 (49.5)	105 (50.5)				
Users	54 (20.6)	30 (55.6)	24 (44.4)				
Alcohol				0.028	0.295 (0.094-0.931)	0.438	0.570 (0.138-2.354)
Nonusers	245 (93.5)	120 (49.0)	125 (51.0)				
Users	17 (6.5)	13 (76.5)	4 (23.5)				
Hypertension				0.004	2.250 (1.293-3.916)	0.574	1.230 (0.632-2.287)
No	188 (71.8)	106 (56.4)	82 (43.6)				
Yes	74 (28.2)	27 (36.5)	47 (63.5)				
Diabetes Mellitus				0.301	3.143 (0.323-30.613)	0.398	2.764 (0.262-29.138)
No	258 (98.5)	132 (51.2)	126 (48.8)				
Yes	4 (1.5)	1 (25.0)	3 (75.0)				
BMI							
<25 kg/m ^2^	135 (51.5)	82 (60.7)	53 (39.3)	0.001*	2.306 (1.405-3.783)	0.034	1.881 (1.050-3.371)
≥25 kg/m ^2^	127 (48.5)	51 (40.2)	76 (59.8)				
OHI				0.000**	3.302 (1.990-5.477)	0.000**	5.189 (2.752-9.784)
Good (0.0-2.00)	132 (50.4)	86 (65.2)	46 (34.8)				
Poor (2.01-6.00)	130 (49.6)	47 (36.2)	83 (63.8)				
Plaque Index				0.000**	2.713 (1.646-4.472)	0.482	1.274 (0.649-2.500)
Slight (0.0-1.39)	130 (49.6)	82 (63.1)	48 (36.9)				
Abundance (1.4-3.0)	132 (50.4)	51 (38.6)	81 (61.4)				

Chi-Square test.

Multivariate logistic regression.

Multivariate logistic regression analysis showed that periodontitis subjects were more likely to have a BMI ≥ 25 kg/m ^2^ (p < 0.001; adjusted odds ratio 1.881; 95% CI: 1.050-3.371). The result were rejected null hypotheses, that there is effect of obesity towards the severity of periodontitis.

The risk of periodontitis was higher in male compare to female (p < 0.001; adjusted OR 6.852; 95% CI: 2.540-18.481), and increased with OHI. Adjusted OR for smoking showed that non-smoking subjects were more prone to periodontitis. The ROC analyses from multivariate logistic regression had an area under the curve of 0.78 (sensitivity 71.3%, specificity 61.7%), determining that it was acceptable using this approach to discriminate between those individuals with healthy or mild periodontitis and severe periodontitis.

**Figure 3.**
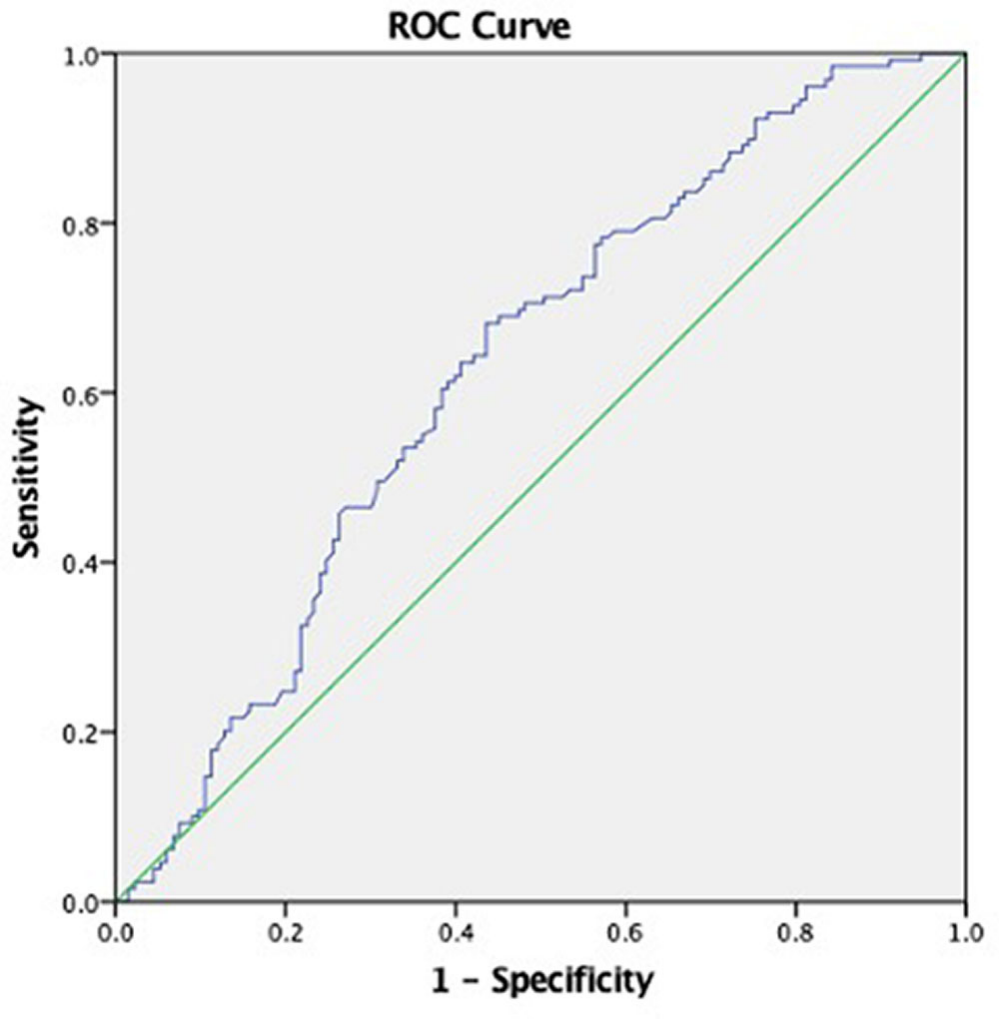
Cut off point body mass index: AUC = 0.629; BMI = 24.785 kg/m ^2^; sensitivity 60.5%, specificity 60.9%.

Despite the fixed value of the Asian cut-off point in BMI, we tried to estimate the cut-off point of BMI in our subjects. Figure 3 shows that the BMI special cut point with the periodontal status, was found in the value of 24.785 kg/m ^2^ (sensitivity 60.5%, specificity 60.9%, AUC 0.608).

## Discussion

The characteristic data ( Table 1) showed that the obesity showed from age 30 and higher in female. This data according to the previous study that the prevalence of obesity was generally higher in female and being maximal between 50-65 years old as in this study 55-66 years old. ^27^ The education in this study, showed no correlation with obesity contrary to study in Korea found that high education were less having obesity prevalence than less educated, but in those study conducted on female only. ^28^

The occupation showed correlation with obesity. Study in Thailand showed that no simple relationship between the occupations and obesity. The obesity rates vary between occupation, also the patterns different for women and men. ^29^

The risk factors for obesity not correlated with alcohol consumption and diabetes mellitus because the smoker and the subjects consume alcohol outnumber subject non-smoker and non-alcohol. Smoking and alcohol consumption and blood pressure related to obesity in previous study. In line with those study, hypertension showed significant result. ^30^

Periodontitis showed correlation with obesity in line with a previous study (in which the age ranged from 25 to 66 years) showed that the prevalence of periodontitis was greater among subjects with obesity and the increased was BMI proposed as potential risk factor for periodontitis. ^15^ Oral hygiene and plaque index ( Table 1) showed no correlation with obesity. This is contrary with the previous study showed that patients with poor oral hygiene tend to suffer from obesity than patients with good oral hygiene. ^31^

Sources of potential bias were that BMI and not body fat distribution, which can be calculated by waist circumference (WC), was measured. Fat distribution varies between woman and men and between race/ethnic groups. ^32^^,^^33^ Participants were taken from three district in central Jakarta which represent mixed ethnic in Indonesia and can be generalized as Indonesian sub population.

The present study covered a slightly wider range of age from 18 up to 66 years old. The highest group with severe periodontitis was in the 45-54 years age group (46.5%). Indonesian prevalence data for periodontitis also showed the biggest prevalence was in 45-54 years age group (77.85%). ^11^

The WHO BMI categories define underweight (<18.5 kg/m ^2^), normal weight (18.5-24.9 kg/m ^2^) and overweight (25.0-29.9 kg/m ^2^), and obese (≥30 kg/m ^2^) while in WHO Asia-Pacific the agreed cut-off for overweight, obese I and obese II category are 23.0 kg/m ^2^, 25-29.9 kg/m ^2^ and ≥30 kg/m ^2^ respectively. ^13^ In the Asia-Pacific area, the BMI cut-off for obesity is 25 kg/m ^2^ rather than the WHO cut-off which is 30 kg/m ^2^. ^34^ We have assumed that the BMI classification for the Asia-Pacific region is more suitable for Asian patients.

The specific cut off BMI in this study of 24.785 kg/m ^2^ (sensitivity 61.1%, specificity 61%, AUC 0.63) shows resemblance to a study by Suvan et al. which has a cut-off point of 24.32 kg/m ^2^ (sensitivity 61.54%, specificity 61.89%). ^15^ Likelihood ratio (LR) in this study was 1.56 (the disease is 1.56 times more likely in individual with the BMI ≥ 24.78 compare to normal weight < 24.78). This exploration by use of the ROC curve confirmed that using WHO Asian-Pacific standard was more suitable in our study rather than BMI 25-29.99 kg/m ^2^ defined for overweight and BMI ≥ 30 kg/m ^2^ for obesity. ^9^ The BMI increased with increasing age and severity of periodontitis. Both the continuous data and the categorical data showed correlations between BMI and periodontitis. This data was in agreement with previous studies among the population aged 28-55 ^14^ and among adults aged 18 to 24 years. ^35^ The clinical parameters of periodontitis (PD, CAL, recession and BOP) presented positive correlations with BMI (p < 0.05). This finding was in line with Zimmermann et al. , a German population cohort study which also found an increased deeper periodontal pocket with higher BMI (OR = 1.6, BMI increase by 5). ^36^

In this study, education level was at the margin of statistical significance (p = 0.053), as in a previous study which showed association with education, tooth loss, and oral hygiene. ^18^ Tooth loss and oral hygiene were not related to increased BMI in this study, may be because the lowest remaining teeth was 14 teeth as an inclusion criteria while in that study, there was no particular inclusion criteria based on tooth loss. Song *et al.* in their study found that tooth loss was considered a potential risk indicator for being underweight in Korean adults. ^37^ Regarding poor periodontal health with obesity, subjects had poor compliance towards oral hygiene, ^16^ and obese patients had an approximately three fold higher number of bacterial species present compared with normal weight controls with total of 23 species. ^36^ In this study, oral hygiene was not related to BMI although oral hygiene is significantly related with the severity of periodontitis.

Although many studies have shown the relationship between BMI and periodontitis ^20^^,^^36^^,^^38^^,^^39^ as in this study, there are some studies which have on the contrary showed no difference between no or mild periodontitis, moderate periodontitis and severe periodontitis. ^18^^,^^40^ Also in another study of BMI and periodontitis in postmenopausal women, those with higher BMI had decreased odds (OR) for having periodontitis compared to participants with normal weight (OR: 0.54; 95% CI: 0.27-0.87) although the obese presented significantly higher clinical attachment loss and gingival index compared to normal and overweight subjects (p < 0.01). ^41^ On the contrary, Puspitadewi et al. in the study of postmenopausal women found no significant correlation between age, BMI, bone density and alveolar bone resorption (p > 0.05). ^10^

Females had a higher BMI compared to male subjects, and according to occupation housewives have the highest BMI compared to employed subjects and students, this corresponds with other studies that have found females to have a higher BMI compared to males. ^18^ Smoking subjects in this study had a lower BMI than non-smoking subjects. Smoking was not correlated with periodontitis, but after adjusting other covariates, a significant correlation was found between non-smokers and periodontitis. This may be due to the low percentage of smokers (20.6%) compared to non-smokers (79.4%) in our study. BMI has multiple risk factors ( Table 2) but after multivariate logistic regression with BMI as dependent factor, only age give a significant result p < 0.001; OR 3.63 (95% CI, 1.86-7.12).

Multivariate regression in role factors in having periodontitis, adjusted OR showed sex, OHI, smoking, and BMI, p < 0.001, OR 6.852 95% CI; p < 0.001, OR 5.189 95% CI 2.752-9.784; p = 0.007, OR 3.803 95% CI 1.449-9.979; and p = 0.034; OR 1.881 95% CI 1.050-3.371 respectively. Together, it means obese male non-smokers with bad oral hygiene are prone to periodontitis.

The odds ratio for BMI was 1.881 (95% CI 1.050-3.371) slightly higher than a previous study where OR was 1.56 (95% CI 1.26-1.92). ^35^ This finding was contrary to a study in Korea by Kim et al. who with multivariate analysis found no association between BMI and periodontitis with BMI ≥ 25, adjusted ratio 0.991 (95% CI 0.806-1.220) but found a significant association between abdominal obesity and periodontitis with an adjusted odds ratio of 1.358 (95% CI 1.003-1.839). ^17^

Mechanism between obesity and periodontitis has potential interlink between the two diseases. Comorbidities related to obesity related to adiposity-association hyperinflammation and cytokine response. Adipocytes secrete hormones and cytokines (adipokines). Normal adipocytes secrete anti-inflammatory adipokines, but hypertrophied adipocytes secrete abnormal adipokine. Macrophage accumulated in adipose tissue, promotes chronic low-grade inflammation in obesity by overproduction of reactive oxygen and nitrogen then increased oxidative stress. Hyperoxidative stress leading to susceptibility to bacterial infection and induce periodontitis or increase periodontitis progression. ^2^^,^^42^ This evidenced of inflammation measured by C-reactive protein which associated with periodontitis and obesity. Dysbiosis of the microbiome in oral and intestinal, immune dysfunction and genetic also play role in etiopathogenesis obesity and periodontitis. ^43^

Metabolic syndrome is a set of risk factor for type 2 diabetes mellitus and cardiovascular disease. The risk factors include impaired glucose tolerance, obesity, pro-inflammatory state etc. Obesity as one of the metabolic syndrome enhance the destructive effects of periodontitis. ^44^

Associations between obesity and periodontitis were found more consistently for visceral than general adiposity, suggesting that visceral fat accumulation measurement may be more strongly associated with periodontitis more than BMI. ^38^ So we suggest for further study including waist hip ratio as a comparing method for BMI measurement.

## Conclusion

This study showed a significant correlation between BMI and periodontitis in Indonesian adults. Therefore, BMI evaluation can be used as a factor for assessing the risk of periodontitis.

## Data availability

### Underlying data

Harvard Dataverse: Raw Data of effect of obesity on risk and severity of periodontitis. https://doi.org/10.7910/DVN/MBVN3O. ^45^

This project contains the following extended data:

• Data_Subject_ObesityResearch.tab (raw per subject data).

Data are available under the terms of the Creative Commons Zero “No rights reserved” data waiver (CC0 1.0 Public domain dedication).

## Consent

Written informed consent for publication of the patient details was obtained from the patients.

## Author contributions

CM: Data Curation, Investigation, Visualization, Writing-Original Draft Preparation, Writing-Review & Editing; EIA: Conceptualization, Recourses, Supervision, Validation, Writing-Review & Editing; SLM: Methodology, Recourses, Supervision; LSK: Formal Analysis, Supervision, Validation; CP: Formal Analysis, Supervision; NS: Data Curation, Formal Analysis.
