# Supplementary material for: Obesity correlated to a higher risk of acquiring periodontitis: a cross-sectional study
Source: F1000Res. 2022 Jan 25;10:643. Originally published 2021 Jul 23. [Version 2] doi: 10.12688/f1000research.53823.2 (PMC8792873; doi:10.12688/f1000research.53823.2)
Supplement: Peer review history

# Peer review: Reviewer response for version 2

**DOI:** 10.5256/f1000research.119885.r120933

**Published:** 2022-01-26

**Authors:** Muhammad Sohail Zafar

**Affiliations:** 1 Department of Restorative Dentistry, College of Dentistry, Taibah University, Medina, Saudi Arabia

Many thanks for responding to the comments.

Is the work clearly and accurately presented and does it cite the current literature?

Yes

If applicable, is the statistical analysis and its interpretation appropriate?

Yes

Are all the source data underlying the results available to ensure full reproducibility?

Yes

Is the study design appropriate and is the work technically sound?

Yes

Are the conclusions drawn adequately supported by the results?

Yes

Are sufficient details of methods and analysis provided to allow replication by others?

Yes

Reviewer Expertise:

Dentistry

I confirm that I have read this submission and believe that I have an appropriate level of expertise to confirm that it is of an acceptable scientific standard.

# Peer review: Reviewer response for version 1

**DOI:** 10.5256/f1000research.57246.r90954

**Published:** 2021-09-15

**Authors:** Muhammad Sohail Zafar

**Affiliations:** 1 Department of Restorative Dentistry, College of Dentistry, Taibah University, Medina, Saudi Arabia

- The title of the study is clear and concise.
- The abstract of the study is adequately written, mentioning the key aspects of the paper.
- The introduction is clearly written with relevant and latest citations. However, the authors should mention the names of proinflammatory cytokines before abbreviating them (Page 3, second paragraph). Furthermore, the introduction should be expanded a bit as it seems to be brief, especially the first and second paragraphs.
- In the methods section, the authors should mention the STROBE checklist as it is an observational study.
- In the methods section, the authors should separate the diagnostic criteria of periodontal disease and obesity by giving them separate sub-headings.
- The authors should also mention the null hypothesis of this study.
- The authors should carefully check the use of abbreviations in the main text, tables, and figures.

Is the work clearly and accurately presented and does it cite the current literature?

Yes

If applicable, is the statistical analysis and its interpretation appropriate?

Yes

Are all the source data underlying the results available to ensure full reproducibility?

Yes

Is the study design appropriate and is the work technically sound?

Yes

Are the conclusions drawn adequately supported by the results?

Yes

Are sufficient details of methods and analysis provided to allow replication by others?

Yes

Reviewer Expertise:

Dentistry

I confirm that I have read this submission and believe that I have an appropriate level of expertise to confirm that it is of an acceptable scientific standard, however I have significant reservations, as outlined above.

# Response

**Published:** 2021-12-29

**Authors:** Elza Ibrahim Auerkari

**Affiliations:** University of Indonesia, Indonesia

Dear Sir, thank you so much for the constructive suggestion in our articles. Hereby we revised the article accordingly. The suggested changes and extensions have been implemented in the introduction, paragraphs 1 and 2, of the revised paper. The strobe checklist has been added in the methods section. also the separate the diagnostic criteria between periodontal disease and obesity by giving them separate sub-headings. The null hypothesis of this study has been added to the methods section and the use of abbreviations has been checked and missing ones added.

# Peer review: Reviewer response for version 1

**DOI:** 10.5256/f1000research.57246.r90953

**Published:** 2021-08-13

**Authors:** Euis Reni Yuslianti

**Affiliations:** 1 Department of Oral Biology, Faculty of Dentistry, Jenderal Achmad Yani University, Cimahi, Indonesia

I think this paper excellent and is an important addition to the literature.

 Please provide more depth exploring in the discussion, first paragraph – as noted before about the correlation between socio-demographic data, oral hygiene and continuous data of body mass index including alcohol, DM, and plaque index.

 Please provide more literature and a deeper discussion about how obesity can effect on risk and severity of periodontitis.

 Linking to the results, please include more detail about the correlation between BMI, obesity, and metabolic syndrome to severity of periodontitis.

 Please add more depth exploring in the discussion related to adipokines that are secreted from fat cells (adipocytes) in obesity which has an important role in regulating metabolic and oxidative stress to periodontitis.

Is the work clearly and accurately presented and does it cite the current literature?

Yes

If applicable, is the statistical analysis and its interpretation appropriate?

Yes

Are all the source data underlying the results available to ensure full reproducibility?

Yes

Is the study design appropriate and is the work technically sound?

Yes

Are the conclusions drawn adequately supported by the results?

Yes

Are sufficient details of methods and analysis provided to allow replication by others?

Yes

Reviewer Expertise:

Bochemistry and Biomolecular, Oral Biology

I confirm that I have read this submission and believe that I have an appropriate level of expertise to confirm that it is of an acceptable scientific standard.

# Response

**Published:** 2021-12-29

**Authors:** Elza Ibrahim Auerkari

**Affiliations:** University of Indonesia, Indonesia

Thank you so much for your kind suggestions for our article.

 Regarding your comment we completed as follows: the aspects of discussion in the first paragraph, have been addressed in the revised paper. More literature and a deeper discussion about how obesity can effect the severity of periodontitis also have been added to the discussion and references of the revised paper. More detail about the correlation between BMI, obesity, metabolic syndrome to the severity of periodontitis, and discussion about adipokines role in regulating metabolic and oxidative stress to periodontitis have been added to the discussion of the revised paper.
